# Metagenome-Assembled Draft Genome Sequence of a Novel Microbial *Stenotrophomonas maltophilia* Strain Isolated from *Caenorhabditis* *remanei* Tissue

**DOI:** 10.1128/genomeA.01646-16

**Published:** 2017-02-16

**Authors:** Janna L. Fierst, Duncan A. Murdock, Chamali Thanthiriwatte, John H. Willis, Patrick C. Phillips

**Affiliations:** a Department of Biological Sciences, University of Alabama, Tuscaloosa, Alabama, USA; b Institute for Ecology and Evolution and Department of Biology, University of Oregon, Eugene, Oregon, USA

## Abstract

*Stenotrophomonas maltophilia* is a Gram-negative aerobic bacterium and emerging nosocomial pathogen. Here, we present a draft genome sequence for an *S. maltophilia* strain assembled from a metagenomic DNA extract isolated from a laboratory stock of the nematode worm *Caenorhabditis remanei*.

## GENOME ANNOUNCEMENT

*Stenotrophomonas maltophilia* is a ubiquitous aerobe found in clinical samples and soil environments (1). It is the most frequent Gram-negative microbe found in hospitals after *Acinetobacter* sp. and *Pseudomonas* sp. (2) and a source of dangerous nosocomial infections (3), due to its genomic repertoire of drug-resistance systems (4) and ability to adhere to plastics and form biofilms (5). *S. maltophilia* has been found with natural isolates of the nematode *Pristionchus* (6) but is a lethal pathogen of *C. elegans* in the laboratory (7). Lethality is strain-specific, and *S. maltophilia* soil isolates result in high *C. elegans* mortality, while the clinical type sample K279a causes low mortality similar to the standard laboratory *C. elegans* food source *Escherichia coli* OP50 (8). *C. remanei* strain PX356 is an inbred population derived from the Caenorhabditis Genetic Center strain EM464 and has been maintained in the laboratory for >50 generations. The *S. maltophilia*-nematode association is an intriguing system for studying host–pathogen interactions and coevolution in a clinically important bacterium.

Sequencing libraries were prepared as described previously (9). Briefly, genomic DNA was isolated from starved L1 *C. remanei* larvae and mixed stage populations with the DNeasy blood and tissue kit (Qiagen) following the *C. elegans* supplementary protocol. Paired-end libraries were constructed with the Nextera kit (Illumina) and size-selected on 2% agarose gels for an average genomic insert size of 180 bp. Mate-pair libraries were constructed by shearing genomic DNA using a Bioruptor sonicator (Diagenode) and purifying with the desalting and concentrating DNA section for the QIAEX-II kit (Qiagen). End repair was performed with the End-it kit (Epicenter). Genomic DNA was biotin-labeled with 1 mM dNTP (4% biotin), and 3-, 5-, and 7-kb fragments were isolated and purified with the QIAEX II kit. Libraries were circularized overnight using T3 ligase (Enzymatics) and T4 ligase buffer. DNA was sheared to 400 bp, and biotin-labeled fragments were isolated with Dynabeads M-280 strepavidin (Invitrogen). All libraries were sequenced as 2 × 101-nucleotide paired-end reads with an Illumina HiSeq instrument.

Assembly of the sequenced libraries with ALLPATHS-LG (10) produced ~18 Mb of sequence data, identified with BLAST (11) to be of nonnematode origin. The Blobology protocol (12) was used to assemble the *S. maltophilia* genome sequence. Briefly, short contiguous sequences were assembled with ABySS (13) and assigned taxonomic origin with BLAST (11). Sequence reads were assigned to 32 species of *Alpha*-, *Beta*-, and *Gammaproteobacteria* with >80% of the microbial sequences originating from *Xanthomonadales* spp.; 14,267,624 *Xanthomonadales* sequence reads were isolated and assembled with ALLPATHS-LG. The resulting genome sequence was 4,602,647 bp (310× coverage; 66.23% GC) contained in two scaffolds.

Functional annotation was performed with the NCBI Prokaryotic Genome Annotation Pipeline (PGAP) (14) and the RAST annotation server (15). The genome contained 4,142 genes and 4,068 coding sequences. Functional annotation identified six rRNAs, 64 tRNAs, four noncoding RNAs, and 34 pseudogenes. Phylogenetic analysis of the 16S ribosomal sequence indicated that this strain of *S. maltophilia* is novel and closely related to *S. maltophilia* ZZ7, isolated from marigold soils (16).

### Accession number(s).

The genome sequence is available from the NCBI GenBank database under BioProject PRJNA248909, BioSample SAMN06040735, and accession number MPSI00000000 (*S. maltophilia* strain SIDR01).
